# The Long or the Post of It? Temporality, Suffering, and Uncertainty in Narratives Following COVID-19

**DOI:** 10.1007/s10912-023-09824-y

**Published:** 2023-11-14

**Authors:** Katharine Cheston, Marta-Laura Cenedese, Angela Woods

**Affiliations:** 1 Institute for Medical Humanities and Department of English Studies, Durham University, Durham, UK; 2 Department of Comparative Literature, University of Turku, Turku, Finland; 3 Centre Marc Bloch, Berlin, Germany

**Keywords:** Long COVID, Post-viral syndromes, Illness narrative, Temporality, Suffering

## Abstract

Long COVID affects millions of individuals worldwide but remains poorly understood and contested. This article turns to accounts of patients’ experiences to ask: What might narrative be doing both *to* long COVID and *for* those who live with the condition? What particular narrative strategies were present in 2020, as millions of people became ill, *en masse,* with a novel virus, which have prevailed three years after the first lockdowns? And what can this tell us about illness and narrative and about the importance of literary critical approaches to the topic in a digital, post-pandemic age? Through a close reading of journalist Lucy Adams’s autobiographical accounts of long COVID, this article explores the interplay between individual illness narratives and the collective narrativizing (or making) of an illness. Our focus on temporality and suffering knits together the phenomenological and the social with the aim of opening up Adams’s narrative and ascertaining a deeper understanding of what it means to live with the condition. Finally, we look to the stories currently circulating around long COVID and consider how illness narratives—and open, curious, patient-centered approaches to them—might shape medicine, patient involvement, and critical medical humanities research.

We start this article with two short extracts that tell a now-familiar story: that of getting a virus and being left with strange, fluctuating, and persistent symptoms:

I am in my early 40s and was generally pretty fit and active, but this [virus] hit me hard. My limbs and head ached, my throat burned and my head was foggy. … After seven days my temperature went up from a fever of 37.7C (100F) to a burning hot 39.4C (103F) and stayed there for 10 days. The pain in my back was agony. … For me, the illness lingered. I couldn’t sleep. I felt nauseous and had horrific abdominal pain. I sweated and shivered all the time. I couldn’t stand up but lying down was painful. … Then came the breathlessness. First from walking up the stairs. Then just lying in bed, it felt impossible to fill my lungs. … On good days I can go for a slow walk—pausing to sit on pavements and fallen trees to catch my breath. I can hold a conversation and pass myself off as fairly normal. On bad days it feels impossible to move from bed. The mattress feels like a ship rolling in a rough sea, my hands shake, my vision blurs, I struggle for breath, my body shivers and vibrates, and every sound cuts through my head like shattered glass. I’ve never experienced anything like it. … After an illness you would expect a steady improvement and a return to normality. Maybe a couple of days or a week in bed - but it’s not happening. Before I got sick I cycled every day, swam twice a week and went hill-walking when I could. Now a slow totter around the park leaves me breathless and exhausted. I can still do things but every action has repercussions. If I empty the whole dishwasher at once I might get a migraine so I do one layer at a time. If I go for a walk, I have to go straight to bed afterwards. If I walk too far I might end up with a fever. Vertigo, brain fog, tremors and heart palpitations come and go as they please. And there’s the constant sinking fatigue—plus a gnawing anxiety because I don’t know when I will get better. And no-one seems to know what is happening in my body. (Adams 2020)

I was about to speak when an intense wave of nausea surged through me. … I wrapped my arms over my stomach and slid down in my seat. By the time we reached campus, half an hour later, I was doubled over, burning hot, and racked with chills. Borden called the campus paramedics. … In the next few days, everything I ate made my abdomen balloon. I radiated heat, and my joints and muscles felt bruised. Every day on the way to classes, I struggled a little harder to make it up the hill behind my apartment. Eventually, I began stopping halfway to rest against the trunk of a tree. One morning, I woke to find my limbs leaden. I tried to sit up but couldn’t. I lay in bed, listening to my apartment mates move through their morning routines. It was two hours before I could stand. On the walk to the bathroom, I had to drag my shoulder along the wall to stay upright. … The lymph nodes on my neck and under my arms and collarbones were painfully swollen. During the day, I rattled with chills, but at night I soaked my clothes in sweat. I felt unsteady, as if the ground were swaying. My throat was inflamed and raw. A walk to the mailbox on the corner left me so tired that I had to lie down. Sometimes I’d look at words or pictures but see only meaningless shapes. I’d stare at clocks and not understand what the positions of the hands meant. Words from different parts of a page appeared to be grouped together in bizarre sentences. … My world narrowed down to my bed and my window. I could no longer walk the length of my street. My hair was starting to fall out. I hadn’t had a period in four months. My mouth and throat were pocked with dozens of bleeding sores and my temperature was spiking to a hundred and one every twelve hours, attended by a ferocious sweat … whenever I overextended myself my health disintegrated. One mistake could land me in bed for weeks, so the potential cost of even the most trivial activities, from showering to walking to the mailbox, had to be painstakingly considered. Sometimes I relapsed for no reason at all. (Hillenbrand 2003)

When stories such as these flooded social and mainstream media from mid-2020, they contradicted initial claims—made, for example, in government policy and by the media—that only two outcomes followed infection with the novel coronavirus SARS-CoV-2: either a mild and self-limiting illness that resulted in full recovery or a severe illness necessitating hospitalization and, in some cases, leading to death. Instead, these stories asserted a third outcome: the uncertain stasis of persistent, potentially severe, and often unexplained symptoms. Of the many descriptive and diagnostic labels in circulation to refer to these symptoms (Garg et al. 2021), we choose to use the “patient-made” term long COVID (Callard and Perego 2021; Perego et al. 2020).

Long COVID is the subject of our first extract, written by Scottish journalist Lucy Adams. The full article is entitled “BBC Correspondent: ‘Long COVID Has Left Me Exhausted for Seven Months” and it was published by the BBC on November 7, 2020. Adams updated her readers in a second article, entitled “Long COVID: Will I Ever Get Better?”, published eight months later alongside the homonymous BBC Panorama documentary (BBC 2021). The question in Adams’s second article is one that will resonate with a large—and growing—number of people: Adams is one of an estimated 2.1 million people in the United Kingdom (Office for National Statistics 2023) and 65 million worldwide (Davis et al. 2023) who, as of January 2023, reported living with the condition.

The second extract is taken from the essay “A Sudden Illness” by United States writer Laura Hillenbrand. Hillenbrand’s sudden illness, however, was not COVID-19: this award-winning essay, published in the *New Yorker* in 2003, details Hillenbrand’s chronic health issues that followed an unnamed (and presumably unidentified) viral infection she contracted on a “Sunday night, March 22, 1987”—33 years, almost to the very date, before Lucy Adams’s illness began just as suddenly. A huge variety of diagnostic labels have been employed over the past century to refer to a particular constellation of symptoms that occur and persist after viral infections. Hillenbrand’s eventual diagnosis, chronic fatigue syndrome (CFS), was coined by the US Centers for Disease Control (CDC) in response to an outbreak of a so-called chronic flu-like illness in Nevada in 1984–1985 (Holmes et al. 1988; Steinbrook 1986), while a 1956 letter published in *The Lancet* proposed “myalgic encephalomyelitis” as an “acceptable” term for what the author described as a “new clinical entity” (Anonymous 1956) that followed a number of outbreaks of a mysterious, *polio-esque* virus across Europe and the US in the 1940s and 1950s. The compound term ME/CFS is now most commonly used to refer to the condition, although debates over diagnostic terminologies continue (National Institute for Health and Care Excellence 2021; Callard 2020).

The novel coronavirus, SARS-CoV-2, clearly poses a new viral threat, and long COVID has also been described as a “new disease” (Atherton et al. 2021). But the phenomena of persistent symptoms following viral infection is not new: as Felicity Callard notes, “a new infectious disease, something that brings a ‘new threat’ into the world, also carries the potential to re-envisage and remake the so-called ‘old’” (Callard 2020, 732). Indeed, distinctions between the so-called new and the so-called old are becoming increasingly blurred: recent publications suggest that around half of those with long COVID are estimated to meet the criteria for ME/CFS (Davis et al. 2023; Twomey et al. 2022), while a growing number of studies are finding distinctive clinical abnormalities that are shared between patients with long COVID and patients with ME/CFS (Haffke et al. 2022; Vernon et al. 2022; Nunes et al. 2022). It may be that Adams’s and Hillenbrand’s accounts not only share narrative similarities but also depict symptoms which—although they followed different viruses and occurred three decades apart on different continents—ultimately reveal a similar (or, perhaps, an identical) constellation of biomedical abnormalities.

Our aim, however, is not to diagnose but to interrogate narrative and its functions in our current pandemic contexts and to probe what narrative might be doing both *to* long COVID and *for* those who live with the condition. What particular narrative strategies were present in 2020—as millions of people became ill, *en masse,* with a novel virus—that have prevailed three years after the first lockdowns? And what can this tell us about illness and narrative and about the importance of this particular kind of analysis? We will examine the connections between individual illness narratives, such as those penned by Lucy Adams, and the collective narrativizing (or making) of an illness and how personal accounts of illness might coalesce into an illness or around a diagnosis. Moreover, in bringing Adams’s accounts of long COVID into dialogue with Hillenbrand’s essay, we consider narrative possibilities and futurities and ask how narratives of long COVID—and our literary critical approaches to them—might shape medicine, patient involvement, and critical medical humanities research.

The first part of this article conducts a critical survey of the small but significant literature on long COVID illness narratives. In order to demonstrate the value of literary close-reading to a broader understanding of long COVID and its narratives, we then return to Adams’s two articles, which we analyze through the lenses of both temporality and suffering. Finally, we look at the stories currently circulating around long COVID, and we conclude by insisting upon the importance and necessity of literary critical approaches to illness narratives.

## Long COVID: Novelty and Narrative

In their highly influential article “How and Why Patients Made Long COVID,” Felicity Callard and Elisa Perego (2021, 2) suggest there “are strong reasons to argue that Long COVID is the first illness to be made through patients finding one another on Twitter and other social media.” We can identify five distinctive features of what Callard and Perego present as a new “patient-made” illness. First, as we have already seen, long COVID names experiences in excess of those first recognized as characterizing SARS-CoV-2—excessive either because of their duration, intensity, or trajectory or because they were qualitatively different symptoms from the fever, shortness of breath, persistent cough, and loss of the sense of taste and smell characteristic of COVID-19. Related to this excess is lack: the fact that long COVID was marked initially by a lack of official clinical recognition, variously understood to result from insufficient investigation of COVID-19 outcomes at a population level, a deprioritisation of the chronic when dealing with the ongoing life-threatening emergency of the acute, and insufficient awareness of and attention paid to the evidence of more complex, longer-term symptoms as it started to emerge. Noting that “the highly contagious nature of COVID-19 discouraged traditional face-to-face consultations” and reduced the opportunities for “rich clinical dialogue,” Rushforth and colleagues see long COVID as an illness which, “perhaps uniquely in recent history, emerged largely without the patient’s clinician acting as witness or sounding-board” (Rushforth et al. 2021, 2). Au and colleagues, in their study of US long COVID patients’ experiences of obtaining medical care during the first 18 months of the pandemic, draw attention less to the absence of “rich clinical dialogue” but rather to patients’ experience of medical “gaslighting” characterized by dismissal, delayed diagnosis, and deferred treatment (Au et al. 2022).

However it is explained or construed, the initial widespread lack of formal and interpersonal clinical recognition are important contextual and contributing factors to two further distinctive aspects of long COVID: the role of patients and their use of social and mainstream media in its making. Patients articulated their ongoing experiences of COVID, shared and mobilized this knowledge through a range of online fora, monitored and reported their symptoms, offered advice and support to others, called for action (Long COVID SOS 2020), and participated in academic, clinical, and policy debates. Patients who were also health professionals (Garner 2020a; Siegelman 2020; Bovo 2021) and public figures (Adams 2020; Harding 2020) had a prominent but arguably not defining role in what was very much a bottom-up phenomenon. Major events, publications, and turning points identified by Callard and Perego (2021)—themselves key protagonists in the patient-making of long COVID—are supported and further elaborated by Miyake and Martin (2021) in their analysis of 1.38 million social media posts over a similar period. What these detailed accounts of activity in 2020 show is the final distinctive feature of long COVID to which we want to draw attention, namely, the speed with which it went from first coinage (by Elisa Perego in a tweet on May 20, 2020) to stabilization as a new “scientific object” (Callard and Perego 2021, 3) in meetings between Long COVID SOS and the World Health Organization (WHO) in mid-August 2020.

What was the role of narrative in the making of long COVID? More precisely, what role does narrative play in scholarly efforts to account for the making of long COVID and subsequently to understand its lived experience? It is intriguing to us that narrative is a term conspicuous by its absence from Callard and Perego’s story of patient-made illness. Patients “started sharing experiences on social media”; produced and published “accounts of suffering” in articles and other “patient-led initiatives”; “challenged” and “anatomized the ‘mild’”; “made incidence data into hashtags”; “brought vivid case studies to wide audiences, expanded knowledge of symptoms, and made demands” (Callard and Perego 2021, 1). They did not, for Callard and Perego, tell stories. Miyake and Martin, by contrast, in their mixed methods analysis of patient discourse surrounding long COVID (including over one million tweets, posts, and blog entries, including hashtags and emojis) place the concept of narrative front and center:

The Long COVID community’s voice had a direct impact on increasing collaboration between patients, researchers and healthcare professionals (HCPs), leading to greater awareness, knowledge and improved health support services for long-term COVID-19 patients. As we continue through further unknowns, it becomes increasingly crucial for every country to begin research at ground level, through listening to patient narratives and understanding these narratives as not just “patient accounts,” but as co-producers of scientific knowledge: indeed, as Nature urges, “they must always give proper consideration to the voices of people with COVID-19 and their representatives, who have done so much to put Long COVID on the health-research and policy agenda.” (Miyake and Martin 2021, 2)

Other major social-scientific studies of patients’ experience of long COVID are divided in their engagement, or not, with concepts of narrative. It is not mentioned by Déguilhem and colleagues in their analysis of symptoms and patient profiles associated with the French hashtag #ApresJ20 (Déguilhem et al. 2022) or in interview-based studies reported by Moretti and colleagues (2022) or Duan and colleagues (2022) with Italian and US patients respectively. It is, however, a major focus—conceptually and methodologically—for Rushforth and colleagues in their large qualitative study of UK long COVID patients’ experience (Rushforth et al. 2021; Ladds et al. 2020, 2021). The imperative across the literature to date has been to identify the continuities and major themes in patients’ accounts of long COVID and the experiences to which long COVID has given rise (including interactions with health services, access to care, participation in patient activism, and so forth). Our suggestion here is that there remains a need to attend in detail, and with the tools of literary close-reading, to patients’ narratives *qua narrative* in order to develop more nuanced accounts of the phenomenology of long COVID and to understand the historical resonances in and of these experiences.

## Temporality

Recalling the excerpts with which we began, we turn to the theme of temporality. Temporality has long been a central focus of illness narrative research (Hyden 1997) and continues to spark imaginative responses and analyses (Wasson 2018); the same is true of work on the phenomenology of illness (Toombs 1990; Leder 2021) and within Disability Studies (Kafer 2013; Samuels 2017). The particular relevance of temporality to any discussion of long COVID is immediately evident in the patient-made label (*long* COVID). Academics have already acknowledged and attended to this temporal aspect: Callard’s essay on “Epidemic Time” (2020) and Jessica Howell’s analysis of the “temporal ‘layering’ of COVID-19 narratives” (2022) are especially relevant. Indeed, it could be said that time and temporality could be among the most prominent themes in the growing qualitative literature on long COVID and that this holds true irrespective of methodology. For example, time was a central theme emerging from Pearson et al.’s (2022) analysis of creative narratives written by people living with long COVID, Moretti et al.’s (2022) analysis of semi-structured interviews, and Miyake and Martin’s (2021) mixed-methods big data analysis of social media posts.

The centrality of time and the temporal is obvious from even the briefest glance at the titles of Adams’s two articles. Entitled “BBC Correspondent: ‘Long COVID Has Left Me Exhausted for Seven Months,’” Adams’s first article quantifies ongoing suffering. Nine months later, the punctuation in the title of her follow-up article “ Long COVID: Will I Ever Get Better?” indicates a shift in focus from indicates a shift in focus from?” indicates a shift in focus from months lost to thoughts of an uncertain future. These are but two articles in mainstream media, yet they are, we argue, particularly revelatory of both an individual and a collective response to a new experience of temporality. Adams’s articles are a patchwork of storytelling mediums (including text, photographs, video clips, and external links) that offer an insight into the patient-making of long COVID, which itself could be said to be a patchwork of stories, interwoven across time and place—an attempt to tease out a common narrative thread, to map possible futures, to locate beginnings and endings.

As with most accounts of long COVID available in the public domain, Adams speaks to her readers largely in real time: “It is July now,” she writes in the second article, creating a temporal connection between author and reader, writing and reading in the same month. But Adams describes a different experience of time; in both of her articles, there is a distinct sense that time is not passing as it should. Adams is frustrated by the lack of linear progress: “After an illness,” she writes in her first article, “you would expect a steady improvement and a return to normality. Maybe a couple of days or a week in bed—but it’s not happening.” Instead of a “steady improvement” or a path to recovery, Adams’s illness experience is fluctuating, measured not in time on a clock but in “good days”—when she can “go for a slow walk” and “hold a conversation”—and “bad days,” when “it feels impossible to move from bed.” As she lurches between the highs of the “good days” and the lows of the “bad days,” it is easy to understand why many with long COVID have likened their experience of illness to that of being trapped on a rollercoaster. For Howell (2022, 218), this metaphor “capture[s] the cyclical and inexorable temporality of illness with COVID-19,” connoting “a frightening and unexpected ride that ends up where one began.” “On a rollercoaster,” Howell argues, “emotions are intensified, but movement is circular,” noting that some with long COVID are using the pun “coronacoaster”—a metaphor which, Howell argues, offers “their fellow sufferers, as well as interested readers, a new way to understand the lived experience of the disease” (Howell 2022, 218).

Adams reveals to her readers an additional temporal dimension to this “coronacoaster.” Her articles are punctuated by the intrusion of memories of the past—her past life, her past self—as well as by concerns and apprehension for the future. “Before I got sick I cycled every day, swam twice a week and went hill-walking when I could. Now a slow totter around the park leaves me breathless and exhausted” (Adams 2020). The differences in her bodily capabilities could not be starker; the break between the sentences displays a visual interruption between “before” and “now.” The photographs Adams includes in her two articles provide a more obvious visual distinction between past and present, before and now. The first article is accompanied, under the title, by a headshot of Adams looking directly at the camera, unsmiling; another photo, featured later in the article, shows Adams before her illness: cycling, smiling. The second article features eight photographs, some of which depict Adams pre-COVID (mountain climbing, on a motorbike) and others are post-COVID (wearing a mask, in hospital); the arrangement of the photographs mixes pre- and post-COVID photographs, so readers bounce between past and present.

Yet just as she and her readers are repeatedly pulled back into the past, so are she and they propelled into the future. Her articles are peppered with worries, concerns, and fears: Adams (2021) writes, for example, that while she is “nervous about getting COVID for a third time,” she is “perhaps more apprehensive that this slog of pain and fatigue will just rumble on.” Apprehension rumbles through both articles; Adams refers to “a gnawing anxiety because I don’t know when I will get better” (2020), and this sense of anxiety also gnaws at her readers. The uncertain future is a constant presence, to which Adams refers repeatedly: to the fact that “no-one seems able to explain how long the symptoms will last or how to get rid of them,” that “no-one could understand what was wrong with me” or that “no-one seems to know what is happening in my body,” and that there was “No end in sight” (Adams 2020; 2021). The effect of these temporal intrusions into the present is a back-and-forth movement to the narrative, which is enacted both textually and visually; as the narrative loops back into the past and then, moments later, darts forward into an imagined, uncertain future, the evocative reading experience could be said to mirror the illness experience. This might, perhaps, go some way to explain why readers were so drawn to these long COVID narratives, which flooded mainstream media from mid-2020, at a time when readers—even those not experiencing persistent symptoms—were all on their personal pandemic rollercoasters, perhaps sharing Adams’s worries that there was *no end in sight*.

Adams, however, is not alone on her “coronacoaster.” In her first article, she describes reading an article in the *British Medical Journal* by a Professor at the Liverpool School of Tropical Medicine. Paul Garner, Adams (2020) tells her readers, was “going through the same pick ‘n’ mix pattern of symptoms” as she was—and, similarly, he was writing about them in a series of posts published on the *BMJ Opinion* blog (Garner 2020a; 2020b; 2020c; 2020d; 2021). Adams appears to view Garner’s posts as a potential road map, offering some idea of when she might start to feel better, and she includes links to his blog posts in both of her articles: an intertextual conversation, of sorts, which readers can follow in just a few clicks. Adams also makes mention, in her earlier article, of the broader conversations occurring, at this particular moment in time, within a newly formed community of people with long COVID: “On Facebook and Slack thousands of people in the UK and abroad have joined long COVID support groups” (Adams 2020). Adams’s articles also appear to have become part of this conversation—and part of this collective sense-making. She wrote in her second article that, following the publication of her earlier piece, “hundreds of people got in touch to offer support. People from Australia, France, North America and the Netherlands” (Adams 2021). These were “messages of comfort and condolence and empathy” (Adams 2021)—but they were also attempts to open a dialogue on behalf of others who were suffering.

There is a dialogic aspect to Adams’s articles, which weave together different voices and different forms of knowledge, including the scientific and the academic alongside the personal. Other voices enter into the written text, such as Tim Spector, professor of genetic epidemiology who, Adams (2020) writes, “says the more we know about COVID ‘the weirder it gets.’” The video included at the start of the second article opens up this dialogue to a plethora of other voices, including Adams’s husband and young daughter and a consultant neuropsychiatrist. These two articles encapsulate key aspects of the patient-making of long COVID, as individual illness narratives are seen to join to form a collective, coherent story of a “new disease” with distinct temporal possibilities. Long COVID has a clearly defined beginning—the start of the pandemic—and Adams’s two articles chronicle a developing scientific consensus. The first emphasizes all that is unknown and uncertain, while the second article outlines the progress science and medicine have made through this new terrain: “long COVID is still new and scientists are trying to work out what causes it in certain people and not others—and how to get rid of it” (Adams 2021). “There are multiple theories,” Adams notes, before going on to list four. Similarly, the articles track the passing of time through the growing population of people affected. Adams cites, in her first article, data from the “COVID Symptom Study app,” which stated that “about 300,000 people in the UK” were reporting symptoms lasting for more than a month (Adams 2020); by the time the second article was published, this figure had risen to 962,000, according to what were, at the time, the “latest figures from the ONS” (Adams 2021).

Interestingly, Adams mentions diagnostic labels that pre-date the pandemic: she comments that her “sick note from the doctor says ‘post viral fatigue after contracting COVID-19’” and the video that forms part of the second article includes a brief clip—filmed, we are told, in June 2020—in which she speaks four words: “post-infectious fatigue syndrome.” Yet Adams rejects the “post-” and chooses instead the patient-made label “long COVID.” There is no indication, in either of her articles, that the other diagnoses she briefly alludes to have far longer histories; there is no acknowledgment in the statistics she cites of those, like Hillenbrand, whose post-viral illnesses are measured in decades rather than weeks. Might these narrative new beginnings—this “new disease”—also hint towards new endings? Does the use of *long* contain within it a promise, or a possibility, that *long* might not mean *too long*?

In the final scene of Hillenbrand’s essay, she sees “a slit of light arc over the houses and vanish behind the trees.” It was, she writes, “the first meteor I had seen since that night” (Hillenbrand 2003)—the night, of course, when her sudden illness began. It is a poignant moment of reflection and acceptance of the circular, cyclic movement of chronic illness—and of finding meaning and beauty within suffering. Adams, in contrast, is still pushing towards the linear trajectory of improvement and recovery, as her use of the present continuous makes clear: “I am gradually recovering,” she writes (Adams 2020). Although the end goal of regained health remains out of reach when she puts the final full stop to her second article, Adams tells readers: “I’m pretty determined to hold on to the idea of myself as a bold adventurer, a mother, a wife, a journalist. Even if for now the mountains are just in my mind” (Adams 2021). The narrative possibilities of this collective illness story—of the patient-making of long COVID—are brought into stark relief when Adams’s articles are read alongside Hillenbrand’s essay. Whatever the similarities may be between the *post-* and the *long*, it is easy to see why Adams might be so determined to imagine different endings to her illness story.

## Suffering

At first glance, Hillenbrand’s longer-form essay, written in considered, literary prose, differs considerably in tone from Adams’s brief series of short articles and the BBC Panorama documentary. Yet both texts tell similar stories of illness: writing about their prolonged sufferings, they attempt to make visible what others cannot know, cannot understand, and very often, are unable to even see. Adams starts her 2020 article by positioning herself as someone who, for many years, has been in the business of sharing people’s stories of suffering, particularly of those touched by chronic pain. The question that animates her is “how to convey their suffering on camera”—*how* to tell these stories so that viewers can *empathize* with someone suffering from “something that was essentially invisible” (Adams 2020). She finds that the best way to do so is to lend the microphone directly to the people, to let them speak and “tell the story in their own words” (Adams 2020). So she resolves to take center stage, as uncomfortable as it may be, in order to tell her own story of prolonged illness and suffering to a country—and a world—still spotlighting the virus itself but not looking at its insidious and inexplicable consequences and the suffering endured by unsuspecting people. The *exhaustion* in Adams’s title is certainly first of all physical; however, as studies have pointed out, it also refers to emotional and psychological exhaustion. It becomes clear that suffering exceeds the lived body and spreads to the person’s whole existence by engendering all sorts of difficult feelings. We focus in this last part of our analysis on both *somatic* suffering, as well as what we call *social* suffering.

Both excerpts conjure from the start experiences of acute and sustained somatic suffering. Right after setting the scene of her story, Hillenbrand tells us that “an intense wave of nausea surged through me” and by the time she had reached her destination she was “doubled over, burning hot, and racked with chills” (Hillenbrand 2003). In the next few days, her stomach seethed while the nausea continued, her temperature skyrocketed at regular intervals and she “radiated heat,” her “joints and muscles felt bruised,” and an inexplicable fatigue stranded her in her dorm room for over three weeks, before she had to drop out of college and move back to her mother’s house (Hillenbrand 2003). Slowly but steadily, other symptoms settled in—swollen lymph nodes, heavy sweating, vertigo, loss of hair, amenorrhea, mouth sores, and weight loss. This constellation of symptoms resonates with Adams’s description, 30 years later, of long COVID: high temperature, muscle and joint pain, and breathlessness: “I couldn’t sleep. I felt nauseous and had horrific abdominal pain. I sweated and shivered all the time. I couldn’t stand up but lying down was painful” (Adams 2020). Sixteen months after contracting COVID-19, Adams (2021) writes, “Most days I wake up in pain and go to bed with pain. I have vertigo, migraines and blurred vision. My joints feel like brittle bone grating on metal.” Underscoring the impact that living in such a painful limbo means, in the short video that precedes the article, we see an edited cut of Adams’s video diary, filmed in close-up from a hand-held camera, with entries spanning from March to October 2020. In the last one, Adams starts the recording by trying to say how long she has been ill. She manages to say “I’ve been ill for eight …” before bursting into tears and cutting off, only to start recording again a little later, her eyes red, her cheeks blotched, her voice still coarse from crying.

The use of visual means enhances Adams’s storytelling. Descriptions of symptoms and suffering in her online articles are mostly factual and curt “because I feel I need to explain what ‘long COVID’ is like” (Adams 2020) to those who do not know or understand it or believe its existence. The use of the images and video excerpts from the BBC Panorama documentary adds to Adams’s articles a more emotive layering, a call to empathize with her and her family as they struggle, like many others, through the rollercoaster of long COVID. Unlike Adams, Hillenbrand’s illustrations of her suffering are lusciously evocative: as if exploding after more than a decade of silence, with brutal vigor they draw on all senses and convey, through metaphors and ample use of active, energetic connotation, the ferocious intensity borne by her feeling body. When the vertigo began in 1993, it was not with a faint spinning but as “violently” as if she were “rolling and lurching, a ship on the high seas,” so much that she had to grab a dresser to hold steady. Sleep seems impossible, as she did not *lie* on the bed “so much as *ride* it as it swung and spun.” The forceful movement is depicted as taking place externally (the room whirls, the furniture moves), outside of herself, yet it is so powerful that her body is fully absorbed by it, even in her dreams, which “took place on the deck of a tossing ship, a runaway rollercoaster, a plane caught in violent turbulence, a falling elevator” (Hillenbrand 2003). No less evocatively, Adams uses the same metaphor of a ship at sea when she first describes her ongoing symptoms: “The mattress feels like a ship rolling in a rough sea, my hands shake, my vision blurs, I struggle for breath, my body shivers and vibrates, and every sound cuts through my head like shattered glass” (Adams 2020).

The physical suffering ebbs and flows over time without ever ceasing. The uncertainty it creates at the physical level—how will my body feel tomorrow, next week, next month, next year?—seeps into everyday life and its activities, which become entangled with (limited, prohibited by) the suffering body. Adams calls it the “psychological impact of not getting better after such a long time” (Adams 2020) and concedes that the reverberations of this indeterminacy are hard to explain. The physical unpredictability infuses daily life with a similar sense of existential precariousness that consumes her: “I can still do things but every action has repercussions” (Adams 2020). Sixteen months later, she takes stock of her situation, which has improved but is still wrapped in uncertainty:

Last year my energy was severely limited. I can do more now but there is still a cap. I still try to push through but every time I do my symptoms pull me up. To some the symptoms might not sound too bad. Having a headache for a day is irritating but having a headache and joint pain for months is difficult to handle … I can either work or walk or play with my kids. I can’t do everything. And I still have to take breaks in between. (Adams 2021)

Maria Victoria Bovo, in her essay “ ‘Long COVID’: Making The Invisible Visible,” similarly describes long COVID as a “relapse-remitting condition in which progress is variable. I move forward, then feel I’m regressing” (Bovo 2021, 1511).

Thus, the suffering body challenges any sense of the normativity of the forward-looking life to which the authors, before their illness emerged, as well as everybody else around them, ascribed. Adams aptly talks of a loss of “spontaneity,” which is also often a given-for-granted ingredient of life. However, working life is one of the paramount examples of this contention with social expectations. For instance, Hillenbrand writes of how her writing career started by taking “only assignments that I could do from home … I did the interviews on the phone from bed. Because looking at the page made the room shimmy crazily around me, I could write only a paragraph or two a day” (Hillenbrand 2003). Years later, Adams shares similar anecdotes: “When I wrote the article last autumn about being seven months in to long COVID, it took me months. I wrote that whole piece while lying in bed, typing on my phone with one finger. When I started writing this article earlier this year my hands were often shaking too much to type” (Adams 2021). Still, Adams is eventually able to agree on a “phased return to work,” although the “new” unreliability of her body and the sequelae of COVID-19 engender anxiety and conflicting emotions about the capacity to follow through:

The first week would mean four hours work spread across a week. It sounds so meagre, yet I was terrified. I had not sat at a computer for 10 months. Brain fog had left me struggling for words, struggling to remember things. I agreed to work on a Panorama about long COVID as part of the phased return. I managed the first week and felt delighted with myself. The next day I woke up with a fever and a terrible pain in my kidneys. (Adams 2021)

In the BBC Panorama documentary, Adams says that going back to filming after not working for such a long time was “nerve wracking” (BBC 2021). The documentary also includes testimonies of several people with long COVID. One of them, Susie Farrelly—“one of around a hundred and twenty thousand healthcare workers with long COVID”—shares her worries about whether she will be able to get back to work (BBC 2021). A nurse by training, Susie is “desperate to go back to the ward, but for now can only do desk work from home” (BBC 2021). Like Adams, she often fails to find the words she needs, especially when she is fatigued. In the documentary, as she talks with Adams over Zoom, she is seized by one such instance of memory loss, and we see her in middle-close up, trying to “pick out” the word that she “see[s] in her brain,” attempting a syllable and letting out a little exasperated scream before closing her eyes to concentrate again, her shoulder tensing and eventually releasing in a sigh as she gives up (BBC 2021). The voice-over reminds us that “one in five people with long COVID struggle with memory, concentration or brain fog” (BBC 2021).

It is the irreconcilable conflict between the reality of daily suffering and social life that brings up another kind of suffering beyond the somatic. Stories about post-viral syndromes like ME/CFS and long COVID acknowledge the difficulties encountered by their protagonists, who live through experiences of which others (including public health bodies) appeared ignorant and for which they did not have a vocabulary. In the BBC Panorama documentary, Ferrelly tells Adams that for some time, she wondered whether she was imagining her illness and pushed herself through the peaks of exhaustion that other people were not able to grasp. This is a salient moment of recognition of shared experience—Adams starts crying because “[Susie’s] story is so like mine, it’s hard to listen to”—and responds with the anecdote of a time she went for a short bike ride that later resulted in being bed-bound with a fever: “Anybody who saw me on the bike ride will be like, ‘Well, she’s totally fine,’ you know, ‘She looks fine’” (BBC 2021). The two women both had to face a dismissal of their physical suffering as unreal, as something psychosomatic or “in their head.” This social suffering is manifested through ugly feelings (Ngai 2007), such as feeling ashamed, feeling as if no one understands, feeling alone, and not knowing what is happening inside their bodies. In particular, despite growing numbers, the first COVID long-haulers struggled with widespread disbelief and incomprehension, which is a reminder of how invisible (until then?) post-viral syndromes had been. The pandemic, it seems, has worsened a phenomenon whereby “even in normal times, persistent mysterious symptoms, combined with an uncertain diagnosis, can be a recipient for stigma” (Moyer 2020). Adams (2020) recalls, “By the time I had been sick for seven weeks, I remember telling my brother I felt ashamed for being off work for so long.” In her second essay, she reiterates the same feeling of shame at being ill: “I feared the judgement of others who could see me going out for a walk and would assume I was recovered. What they couldn’t see was the payback afterwards. The fact I would have to go straight to bed or would most likely be punished with a blinding headache, fever or loss of vision later in the day” (Adams 2021). Paul Garner similarly writes of how long COVID sufferers were received with incredulity: “I spoke to others experiencing weird symptoms, which were often discounted by those around them as anxiety, making them doubt themselves” (Garner 2020a). Hillenbrand’s account is no less filled with the despair and the shame of being stigmatized, doubted, disbelieved, and dismissed. Like many of the participants in the most recent long COVID studies mentioned above (Moretti et al. 2022; Au et al. 2022), Hillenbrand, too, points to the twofold lack of support—medical and social, and how the lack of a clear diagnosis has repercussions on how the social world (friends, family, etc.) sees us: “People told me I was lazy and selfish. … I was ashamed and angry and indescribably lonely” (Hillenbrand 2003).

The interlacing of feelings of isolation at being trapped in a room and “left behind” while “outside, the world went on” (Hillenbrand 2003) and simultaneously of shame and being a useless imposter is a leitmotif of post-viral stories that often include bouts of depression and suicidal thoughts (Moretti et al. 2022, 9). Indeed, Hillenbrand’s close encounter with attempted suicide is not due to the illness itself (or only to that) but to the escalation of various forms of suffering, including uncertainty and invisibility. Would her illness story have sounded different had she read an account like her own? Would her suffering have been lessened by contact with others who had, or had had, her same symptoms? In the second half of the 1980s, connecting with strangers on the basis of a shared “sudden” illness was much less possible, and it seems like even the doctors who provided Hillenbrand with a long-awaited diagnosis did not consider putting her in touch with other similarly-affected patients. By contrast, Adams in 2020 speaks of having a “eureka moment” when she read Paul Garner’s account of a similar symptomatology: “I cried with relief.” Being in touch with him for months provided her with help and support. When he tells her that he is feeling better, she gets “a massive boost”: “If he could get better I figured that meant I could too” (Adams 2021).

Since the beginning of the pandemic, the scarce response of public institutions and their contradicting information have given rise to Facebook and Twitter groups, where sufferers of long COVID share their stories and symptoms and bear witness to an unsettling (and upsetting) institutional silence and disbelief (Moretti et al. 2022; Au et al. 2022; Rushforth et al. 2021). As Adams (2020) writes, “people on the long COVID Facebook group are struggling for medical and psychological support. Many feel they are not being listened to.” Hence, it is through social media platforms that long COVID patients have found their listeners, support, and information. Adams again, among others, personally witnessed this coming together outside of institutions: “Most are struggling to get support. … *For those of us on the Facebook Long COVID Support group, this is one of the only places to get help*. When my hair started falling out by the fistful in the summer, other members explained the same was happening to them” (Adams 2020; emphasis added). The sufferers’ tweets and Facebook posts are the “small stories” that made long COVID (Callard and Perego 2021; Miyake and Martin 2021; Déguilhem et al. 2022) and that contribute to creating an archive that challenges invisibility, as well as the linear emplotment of the “Recovery Narrative” (Woods et al. 2022, 221). The multi-media quality of Adams’s long COVID narrative—the published essays, the videos, the photographs—opens further avenues when we also take into account the multiplicity of voices that are juxtaposed in order to “make” her narrative. Significantly, the BBC Panorama documentary features a carrousel of faces and home-made videos of long COVID patients sharing their individual illness experiences, which punctuate Adams’s investigation at different moments. Together with her own testimony and the experts’ statements, this polyphony of voices coalesce to create the collective story of a community—a *collective* illness narrative

Yet, as much as stories of long COVID have crossed the threshold of invisibility thanks to essays like Adams’s, Garner’s, and many others, their presence across countries is not homogenous, thus implying that acknowledgment of long COVID is still slow to come in certain spaces. For instance, whereas there has been significant media coverage of long COVID in the UK, and long-form publications/essays of personal testimonies abound in the UK press, personal narratives are still at the margins of public discourse in other countries. These variations in treatment within the public space mean that patients’ stories are received differently (whether by physicians, family members, co-workers, or institutions) and thus create unequal affective responses at the personal, community, and institutional levels. But does (hyper)visibility lead to more positive experiences of (and/or access to) healthcare, and/or support from the wider public? The tension between invisibility and (hyper)visibility of long COVID (and its narratives) raises questions about the weight of different cultural pandemic responses and how they have shaped such narratives. Stories of long COVID are entangled with the *grands récits* of COVID-19 and their idiosyncratic figurations in different geographical spaces; they also mirror systemic (and epistemic) obliteration depending on the intersectional power dynamics they are embedded in. Within these structural and cultural variations, long COVID narratives and their reception are also bound to evolve in unscripted, open-ended ways.

## Conclusion

This paper differs in many ways from those with which it is in conversation. The most obvious difference is methodological: we have not attended to a large dataset, whether of 20 or well over a million different accounts of long COVID. Our choice to focus, in detail and depth, on just one person’s experiences—as expressed in two articles and through a variety of media—augments existing methods by insisting upon the value of a distinctly literary critical approach to illness narratives *qua narrative* and the insights this approach can illuminate—especially in cases, like long COVID, where an illness is poorly-understood from a biomedical perspective. To acknowledge that Adams’s articles can be seen as, in some sense, exemplary of the patient-making of long COVID is not to deny the structural and social conditions that made it so. Adams, speaking from multiple positions of privilege, is just one of millions of people living with long COVID, most of whom lack the journalistic platform to share their experiences with such a wide (and public) audience. Countless stories remain unheard due to health inequalities deepened by intersectional issues such as race and class—and we welcome future research that seeks out these narratives and brings them to the forefront.

Our analysis of Adams’s illness narrative coalesced around temporality and suffering, which, in and of themselves, are by no means novel topics in the literature. We approached these not as distinct themes—to be compared across accounts and, perhaps, divided into relevant subthemes—but rather as lenses through which to explore, in as full a manner as is possible, the impacts that long COVID is seen to have had on Adams. As such, we attended to the symptomatic and the affective; to all in between, and besides; and to the ways in which these narrative threads are intertwined. We also highlighted the dialogic aspects of Adams’s articles and the ways in which these mapped into the patient-making of long COVID. As such, our analysis held both the individual and the collective, examining the first-person expression of illness experience alongside the means through which those living with illness turn to each other to make storied sense out of their collective suffering. Moreover, in bringing Adams’s two articles into conversation with Hillenbrand’s personal essay, this paper could be said to replicate the “lively exchanges” that Callard and Perego (2021) note were occurring between those with long COVID and those with ME/CFS from the early days of the pandemic—but which are often absent from the literature on long COVID (Pearson et al. 2022; Mullard et al. 2023). Thus, our analysis shows continuities and intersections that gesture toward developing research on collective illness narrative that is more attuned to matters and forms of the communal—the interpersonal, contextual, cultural, and structural within illness experience.

Throughout all of the above, our aim has been to open up Adams’s narrative—to a wider readership, and to a deeper understanding of the insights it communicates about what it means to live with long COVID. We have not sought to impose narrative closure upon it, or to subsume it to the service of an overarching argument (about, for example, the nature of the diagnosis it details). In the first two years of the pandemic—during which Adams wrote and published her articles—there appeared to be an awareness of the need for such an open and curious approach, which centered the patient voice, and treated those living with long COVID as experts of both the symptoms they experience, and of the situation in which they find themselves (Miyake and Martin 2021; Anonymous 2020).

As we write this, three years after the first lockdowns were imposed, the story appears to be changing. In December 2022, journalist Natalie Shure asked, in the *New Republic,* whether “We Might Have Long COVID All Wrong”—whether this might not be (as we read in Adams’s articles) a new disease caused by a novel virus, but instead might be “driven by psychosocial distress” or “triggered by the trauma of the pandemic itself” (Shure 2022). In January 2023, the authors of a comment, published in *The Lancet Respiratory Medicine*, similarly claimed that “a new paradigm is needed to explain long COVID” (Saunders et al. 2023). They propose a causal model in which biological, social, experiential, and psychological factors interact, and they include “available/legitimised illness narratives” as part of this causal model (Saunders et al. 2023). This is ambiguous—and the authors provide no elucidation—but the belief that illness narratives can be contagious or harmful is not novel (just as the constellation of symptoms of long COVID was no novelty to those who suffer from post-viral illnesses) (Showalter 2013). The authors of a 2008 paper on Gulf War Syndrome (GWS)—a contested condition naming persistent symptoms in veterans of the 1991 Gulf War—argued that “the transmission of rumour was a significant part of the very construction of the condition itself” (Cohn et al. 2008). The cause of GWS was ultimately (and recently) determined to be neither narrative nor rumor, however, but a gene-environment interaction, in which veterans with a weak variant of a particular gene were predisposed to experience chronic symptoms following low-level exposure to a nerve agent (Haley et al. 2022). Those living with GWS—whose testimonies were cited in the 2008 paper—were right all along: “something out there in the Gulf” (Cohn et al. 2008) really had made them ill. “It is crucial that those with [long COVID] are listened to in a way that, tragically, people with ME/CFS were not,” wrote the anonymous author of a *Nature* editorial (Anonymous 2020). This is crucial indeed, and we must also ask: had Hillenbrand’s narrative been attended to with appropriate urgency, might Adams’s accounts have existed at all? These are cautionary tales: the literary critical approach that we have illustrated in this paper—which attends to narrative as a crucial insight into illness experience and analyses without imposing an ending or explanation—has never, it could be said, been more urgently needed.
